# Computer-Based Decision Tools for Shared Therapeutic Decision-making in Oncology: Systematic Review

**DOI:** 10.2196/31616

**Published:** 2021-10-26

**Authors:** Alan Yung, Judy Kay, Philip Beale, Kathryn A Gibson, Tim Shaw

**Affiliations:** 1 Research in Implementation Science and eHealth Faculty of Medicine and Health The University of Sydney Sydney Australia; 2 Human Centred Technology Cluster School of Computer Science The University of Sydney Sydney Australia; 3 Concord Cancer Centre Concord Repatriation General Hospital Sydney Australia; 4 Department of Rheumatology Liverpool Hospital, Ingham Research Institute University of New South Wales Sydney Australia; 5 Sydney Catalyst Translational Cancer Research Centre The University of Sydney Sydney Australia

**Keywords:** oncology, cancer, computer-based, decision support, decision-making, system, tool, machine learning, artificial intelligence, uncertainty, shared decision-making

## Abstract

**Background:**

Therapeutic decision-making in oncology is a complex process because physicians must consider many forms of medical data and protocols. Another challenge for physicians is to clearly communicate their decision-making process to patients to ensure informed consent. Computer-based decision tools have the potential to play a valuable role in supporting this process.

**Objective:**

This systematic review aims to investigate the extent to which computer-based decision tools have been successfully adopted in oncology consultations to improve patient-physician joint therapeutic decision-making.

**Methods:**

This review was conducted in accordance with the PRISMA (Preferred Reporting Items for Systematic Reviews and Meta-Analyses) 2020 checklist and guidelines. A literature search was conducted on February 4, 2021, across the Cochrane Database of Systematic Reviews (from 2005 to January 28, 2021), the Cochrane Central Register of Controlled Trials (December 2020), MEDLINE (from 1946 to February 4, 2021), Embase (from 1947 to February 4, 2021), Web of Science (from 1900 to 2021), Scopus (from 1969 to 2021), and PubMed (from 1991 to 2021). We used a *snowball* approach to identify additional studies by searching the reference lists of the studies included for full-text review. Additional supplementary searches of relevant journals and gray literature websites were conducted. The reviewers screened the articles eligible for review for quality and inclusion before data extraction.

**Results:**

There are relatively few studies looking at the use of computer-based decision tools in oncology consultations. Of the 4431 unique articles obtained from the searches, only 10 (0.22%) satisfied the selection criteria. From the 10 selected studies, 8 computer-based decision tools were identified. Of the 10 studies, 6 (60%) were conducted in the United States. Communication and information-sharing were improved between physicians and patients. However, physicians did not change their habits to take advantage of computer-assisted decision-making tools or the information they provide. On average, the use of these computer-based decision tools added approximately 5 minutes to the total length of consultations. In addition, some physicians felt that the technology increased patients’ anxiety.

**Conclusions:**

Of the 10 selected studies, 6 (60%) demonstrated positive outcomes, 1 (10%) showed negative results, and 3 (30%) were neutral. Adoption of computer-based decision tools during oncology consultations continues to be low. This review shows that information-sharing and communication between physicians and patients can be improved with the assistance of technology. However, the lack of integration with electronic health records is a barrier. This review provides key requirements for enhancing the chance of success of future computer-based decision tools. However, it does not show the effects of health care policies, regulations, or business administration on physicians’ propensity to adopt the technology. Nevertheless, it is important that future research address the influence of these higher-level factors as well.

**Trial Registration:**

PROSPERO International Prospective Register of Systematic Reviews CRD42021226087; https://www.crd.york.ac.uk/prospero/display_record.php?ID=CRD42021226087

## Introduction

### Background

As patients continue to play a more active role in the management of their health, the person-centered model of care has been promoted as a strategy to improve the quality of health care systems [1]. Along with ensuring that all clinical decisions are guided by the patient’s values, the goal of the person-centered model is to respect and respond to the individual’s preferences and needs. This motivates physicians and patients to coordinate their activities, share information, and reach shared therapeutic decisions [2]. This review takes a person-centered approach for the important and challenging case of consultations involving patients with cancer. Patients have come to expect their treating physicians to explain the benefits, as well as the risks, of the therapies recommended to them. Furthermore, patients prefer to be engaged in the therapeutic decision-making process [3,4], except when they are very ill [5,6], rather than permitting their physicians to choose therapies for them. Patients may also want to be given the chance to consider their options and to choose between accepting or refusing a therapy.

Medical consultations in oncology are a multipart process that involves shared decision-making between the patient and the physician. Bomhof-Roordink et al [7] have articulated this process in their model of shared decision-making. A physician starts the anticancer treatment recommendation process by learning about the patient’s preferences, before or during consultations, which they need to consider along with the evidence of efficacy of each potential treatment option. Next, the physician needs to engage the patient in reviewing the potential benefits and risks of the key therapeutic choices available. After collaboratively and carefully examining the situation, the physician provides treatment recommendations. However, the ultimate course of action may be chosen by the patient alone or by the physician when the patient does not want to decide [7].

As the choice of diagnostic modalities and therapies grows, the clinical decision-making process has become extremely complex [8]. Faced with large volumes of fragmented information, physicians must reconstruct, identify, and consider the portion of information that they share with their patients. In addition, physicians need to decide how to best inform their patients and obtain their consent [9]. Hence, physicians need clinical information that is organized and presented in a way that is easy for them to interpret and share in discussions with their patients. Once physicians have determined what they need to share, they need to be able to show the relevant information to their patients in such a way that the patient can understand the meaning of the different benefits and risks of each therapeutic choice [5,10]. When physicians can summarize information that is relevant to patients’ diseases and their survival, explain highly uncertain situations, and manage their interactions with patients well, then patients can more easily understand their physicians’ recommendations and choose their preferred therapy or care pathway. This step establishes the foundation for informed consent in shared therapeutic decision-making.

With the intention to support patients, as well as physicians, in this challenging therapeutic decision-making process, paper-based decision tools have been developed [8]. They have been designed to enhance patient-physician communications and interactions. In addition to the incorporation of research results, for example, evidence from clinical trials, paper-based decision tools inform both physicians and patients of the risks, benefits, and outcomes of the available therapies [6,11,12]. Furthermore, paper-based decision tools have a long tradition in supporting clinical decision-making. They have been shown to improve patients’ knowledge, accuracy of perceived potential risks, understanding of prognosis, treatment goals, and health outcomes [8]. Moreover, in practices where paper-based decision tools are used, they are well accepted [11]. However, paper-based decision tools can be difficult to update when new therapies are rapidly being developed and adopted. Furthermore, increasing the use of genetic testing and the introduction of advanced molecular medicine in routine clinical practice has generated an expanding body of knowledge that increases the complexity of the decision-making process [2]. Thus, it is recommended that physicians and patients use computer-based decision tools to improve the process outlined above [2].

Hunt et al [13] defined a computer-based decision tool as follows: “any software designed to directly aid in clinical decision-making in which characteristics of individual patients are matched to a computerized knowledge base for the purpose of generating patient-specific assessments or recommendations that are then presented to clinicians for consideration.”

Research to create computer-based clinical decision tools has a long history. For example, as far back as 1973, Shortliffe et al [14] published a paper on this topic. Shortliffe [15] believed that with computer-based decision tools, knowledge can be integrated and disseminated to physicians. Similarly, computer-based decision tools may aid in packaging relevant clinical information and therapeutic choices for presentation to individual patients [16]. They may also simplify patient-physician communications [8]. On the basis of these perceived benefits, several computer-based decision tools have been developed to assist therapeutic decision-making during oncology consultations [17].

For example, Shortliffe et al [18] developed a computer-based decision tool to guide physicians treating patients with cancer. The technology consists of a computer user interface that enables physicians to review patients’ historical data and test results, enter new information about patients, and query the computer system for anticancer therapy recommendations. The implemented computer technology was initially based on the IF-THEN rule algorithm: for example, “IF: there is evidence of disease extension THEN: refer the patient to lymphoma clinic” [18]. However, more recently, computer-based decision tools have been redeveloped for oncology consultations by applying artificial-intelligence–based machine learning software technologies to improve the accuracy of the recommended anticancer therapies [16].

It is unclear at what level computer-based decision tools are adopted by oncology physicians. There have been a small number of reviews about computer-based clinical decision tools [19-21]. Pawloski et al [21] reported patients’ outcomes from a treatment delivery viewpoint. Beauchemin et al [20] described decision tools broadly and included nursing care delivery in their study. In contrast, Mazo et al [19] provided an overview of decision tools for breast cancer. However, none of the reviews addressed physicians’ propensity to adopt computer-based decision tools during oncology consultations. The aim of this review is to identify and categorize the factors that influence physicians’ propensity to adopt computer-based decision tools in oncology consultations by using the Clinical Adoption Framework (CAF) [22,23].

### Conceptual Model

**Figure 1:**
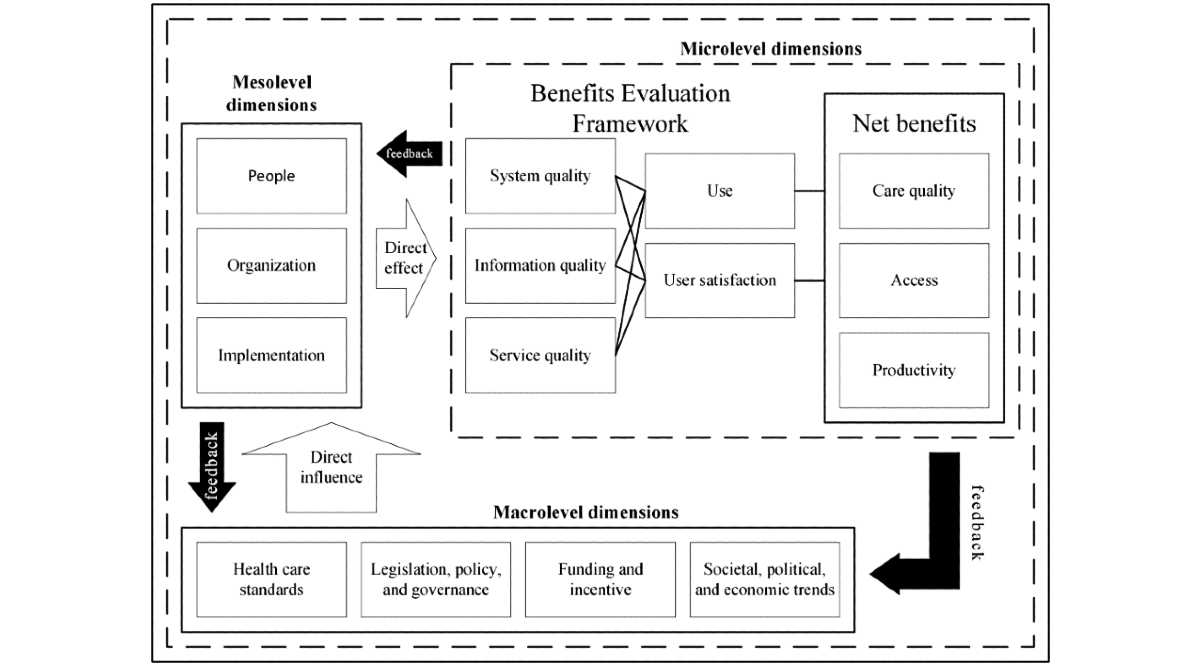
Clinical Adoption Framework with the micro-, meso-, and macrolevel dimensions, which could influence the successful adoption of health information systems, and the associated conceptualized feedback loops [22,24].

The CAF, as shown in Figure 1, is an extension of the Benefits Evaluation Framework (Canada Health Infoway), which was adapted from the DeLone and McLean information system success measurement model, as cited in the study by Lau et al [22].

Conceptually, the CAF is made up of micro-, meso-, and macrolevels. At the microlevel, the focus is on the dimension of quality, which measures success factors such as information completeness, accuracy, relevance and comprehension, system features, performance, security, responsiveness, support services, and leadership; user behavior, intention to use the technology, and user satisfaction; and net benefits, which refer to patient safety, risk, effectiveness, compliance, health outcomes, efficiency and capability, cost and savings, availability and access to services, and patient and clinician participation [24].

The mesolevel dimensions directly influence microlevel users’ propensity to adopt the technology. It addresses people’s characteristics and their expectations, roles, and responsibilities; technology system and organizational fit, strategy, culture, structure or processes, information infrastructure, and return on value; and implementation stages, project management approaches, and technology fit with present and future operations [24].

The macrolevel dimensions directly affect the mesolevel factors, which in turn affect the success of adoption at the microlevel. At the macrolevel, governance, legislations, regulations, and policies; health care and professional practice standards; funding and incentive payments; and trends in public expectations as well as sociopolitical and economic climates with respect to technologies and the whole health care system influence adoption [24].

In addition, as indicated in Figure 1, there is a feedback loop at each level of the CAF. The results of each level are fed back to higher and lower levels of the conceptual model, that is, the outcomes of microlevel factors influence the meso- and macrolevel factors. Similarly, mesolevel factors influence higher macrolevel and lower microlevel factors, and macrolevel factors affect mesolevel factors [24]. Consequently, the CAF represents a technical, social, political, and economic system that must contend with constant internal and external forces that dynamically affect propensity to implement and adopt computerized information systems in health care settings.

The research questions are as follows: (1) What is the extent of adoption of computer-based decision tools in oncology consultations? (2) Is there a difference in levels of adoption by country and period? (3) What factors may have influenced the adoption of the technology? (4) What are the lessons learned to improve adoption of the technology?

## Methods

This systematic review was registered on PROSPERO (CRD42021226087), the International Prospective Register of Systematic Reviews [25].

### Search Strategy and Inclusion Criteria

This study was conducted in accordance with the Cochrane Handbook for Systematic Reviews of Interventions [26] and followed the PRISMA (Preferred Reporting Items for Systematic Reviews and Meta-Analyses) 2020 checklist, guidelines, and statements [27]. In addition, with the assistance of medical sciences librarians, the search strategy was constructed by applying the PICOC framework [28,29]:

- P (population): only physicians treating patients with cancer were included. Other clinicians such as nurses, pharmacists, or supportive care professionals were excluded.
- I (intervention): only computer-based decision tools used to assist oncology consultations were included. Paper-based tools or digital tools such as websites that are used solely and independently by patients who seek information outside consultations with their treating physicians were not included.
- C (comparison): usual care, which means health care based on traditional paper pamphlets, video recordings, or using standard data collection in electronic health record systems.
- O (outcomes): adoption of the technology for use during oncology consultations, that is, physicians use the information provided by computer-based decision tools as part of their routine medical practice to deliver oncology care.
- C (context): assisting shared decision-making during the selection of anticancer therapy, that is, physicians and patients use the information provided by the technology to collaborate and discuss the benefits and potential harms of each treatment option before agreeing on a final treatment plan. In this context, use of the technology does not mean only the physician needs to physically operate or view information on the computer screen. The physician may provide access to the technology to the patient or another care provider to assist the patient enter personal information or understand the information provided. The physician can then use the additional information provided by the patient to facilitate discussions and decision-making during the consultation.

On February 4, 2021, 1 reviewer (AY) used the OvidSP platform (Health First) to search the following databases: Cochrane Database of Systematic Reviews (from 2005 to January 28, 2021), Cochrane Central Register of Controlled Trials (December 2020), MEDLINE (from 1946 to February 4, 2021), and Embase (from 1947 to February 4, 2021). In addition, on the same day, the databases of Web of Science (from 1900 to 2021), Scopus (from 1969 to 2021), and PubMed (from 1991 to 2021) were searched. After relevant articles were selected for inclusion in this review, the reference list and citations of each article were inspected for additional articles. The *snowball* search was conducted using Scopus and Google Scholar. Further searches for relevant articles were conducted by browsing the *BMC Medical Informatics and Decision Making* journal website, along with searches of gray literature websites [30-33]. The detailed Boolean expressions of the search strategy are provided in Multimedia Appendix 1.

### Study Selection

A single review author (AY) removed duplicates and screened the titles and abstracts of all retrieved articles for relevance in accordance with the criteria of the research questions. Similarly, another 2 review authors (JK and TS) independently assessed the eligibility of a randomly selected sample of articles from a subset of the retrieved articles to judge their eligibility for inclusion or exclusion in the review. Disagreement among the 3 review authors was resolved through discussion.

First, guided by the evidence-based medicine pyramid [34], articles that used a study design within the categories of randomized controlled trials, cohort studies, case-control studies, and case series or reports were included for review, whereas articles that were published as conference papers or abstracts, protocols, commentaries, editorials, letters, or opinions were excluded because of their perceived low quality. No limitation on language was imposed. For articles that were not published in the English language, attempts were made to translate them into English by using a web-based translator [35]. Second, studies that met the following key criteria were included: (1) the study was conducted in an oncology consultation setting, (2) it involved distinct real-world computer-based decision tool use by oncology physicians, (3) a computer-based decision tool assisted patient-physician communications to share information and to agree on an anticancer therapy; and (4) the elements of the effectiveness of a computer-based decision tool in oncology consultations were reported.

### Data Extraction

A data extraction spreadsheet to capture study information was developed a priori by 3 reviewers. The selected studies were then screened by 1 review author, and relevant qualitative data were extracted. The spreadsheet was populated in accordance with the requirements of the review questions. As more experience was gained with data extraction, the review authors iteratively adjusted the required variables in the spreadsheet. The final set of data variables required to answer the review questions was as follows: study; study design and participant sample size; computer-based decision tool versus comparator; clinical setting context and country; primary objective; and study outcomes (Table 1).

**Table 1:** Overview of the included studies, ordered with the most recent first (N=10).

Study	Study design and participant sample size	Computer-based decision tool versus comparator	Clinical setting context, country	Primary objective	Study outcomes^a^
Wyatt et al [36], 2019	Pre- and postsurvey patients (n=290), postsurvey patients (n=447)	TakeTheWind versus no comparison	Breast cancer clinic (n=1), United States	To assess utility, ease of use, and impact of decision tool	*Patients preferred shared decision-making* and written material, disliked tablet computers, and had trouble navigating and accessing the tool.
Yao et al [37], 2019	Longitudinal, prospective before-and-after study; CDT^b^-arm patients (n=63), surgeons (n=2); UC^c^-arm patients (n=57), surgeons (n=3)	In-visit decision aid versus UC	Breast surgery clinics (n=5), institution (n=1), United States	To measure impact on knowledge, preferences, and involvement	*Patients had more discussions regarding their treatment with surgeons and had less surgery.* (Anxiety, distress, fear, quality of life, and concerns regarding body image were unchanged) compared with UC.
Cuypers et al [38], 2019	RCT^d^; CDT-arm hospitals (n=9), UC-arm hospitals (n=9), academic medical center (n=1)	Prostaat versus UC	Prostate cancer hospitals (n=18), academic medical center (n=1), the Netherlands	To understand implementation and use of CDT	*Improved physician-patient communication about preferences and values*
Raj et al [39], 2017	Controlled before-and-after study; before-implementation patients (n=80), after-implementation patients (n=134)	COMBAT versus paper	Pain management at outpatient cancer clinic, Norway	To evaluate improvement in pain management	(No change in physicians’ behavior and no improvement in pain management)
Yao et al [40], 2017	Prospective pre-post study; CDT-arm patients (n=97), UC-arm patients (n=114)	In-visit decision aid versus UC	Breast surgery at hospitals (n=3), United States	To examine effects on shared information and treatment choice	*Higher knowledge levels in the CDT group than in the UC group*
Miles et al [41], 2017	Mixed-methods randomized trial; patients (n=13)	Openclinical versus no comparison	Colorectal cancer outpatient oncology department, United Kingdom	To examine acceptability, usefulness, and areas of improvement	*CDT was accepted and found useful by patients* but needed improved presentation of information.
Henton et al [42], 2017	Usability study; patients with prostate cancer (n=7), patients with colorectal cancer (n=7)	SEER*CSC^e^ versus no comparison	Prostate and colorectal cancer centers (n=4), United States	To understand patients’ information needs and preferences	CDT lacked features to facilitate patient-physician discussions and was time consuming for data entry.
Morgan et al [43], 2015	Prospective study; patients (n=25)	Morgan versus no comparison	Breast cancer center, Canada	To assess satisfaction and knowledge retention	*Knowledge retention was high, and patients were highly satisfied.*
Siminoff et al [44], 2006	RCT; physicians (n=58), patient-physician pairs (n=405)	Adjuvant! versus UC pamphlet	Breast cancer oncology practices (n=14), United States	To examine impact on treatment decisions and practice	CDT added 5 minutes to total consultation time *and was found more useful than a pamphlet.*
Peele et al [45], 2005	RCT; physicians (n=56), CDT-arm patients (n=250), UC-arm patients (n=182)	Adjuvant! versus UC pamphlet	Breast cancer practices, academic (n=5), community-based (n=9), United States	To examine impact on women’s adjuvant therapeutic decision	*Fewer women with low tumor severity chose adjuvant therapy.*

^a^To represent the key outcomes of each study, the following formatting has been adopted: *italic text* represents positive outcomes, normal text represents negative outcomes, and normal text within parentheses represents neutral outcomes.

^b^CDT: computer-based decision tool.

^c^UC: usual care.

^d^RCT: randomized controlled trial.

^e^SEER*CSC: Surveillance, Epidemiology, and End Results Cancer Survival Calculator.

### Risk-of-Bias Assessment

Using the Cochrane risk-of-bias tools for randomized controlled trials and nonrandomized studies, 1 review author assessed the risk of bias of the included studies [26]. The tool for randomized controlled trials [46] assesses studies on each of these 6 domains: (1) randomization processes, (2) identification or recruitment of participants into clusters, (3) deviations from the intended intervention, (4) missing outcome data, (5) measurement of the outcome, and (6) selection of the reported result. The tool for nonrandomized studies [47] assesses studies on each of these 7 domains: (1) due to confounding, (2) selection of participants into the study, (3) classification of intervention, (4) deviations from the intended intervention, (5) missing data, (6) measurement of outcomes, and (7) selection of the reported result. Finally, the judgment in each domain is carried forward to an overall risk of bias for each study. The tools highlighted some risk of bias in all the selected studies.

### Data Synthesis

The articles included in this study reported a high diversity of functionalities and features of computer-based decision tools. Therefore, the reported outcomes of the studies were grouped according to the dimensions of the CAF [22]. The results within each group were subsequently assessed and combined into a common set of factors that directly affect physicians’ propensity to adopt computer-based decision tools in oncology consultations.

## Results

### Search Results and Study Characteristics

**Figure 2:**
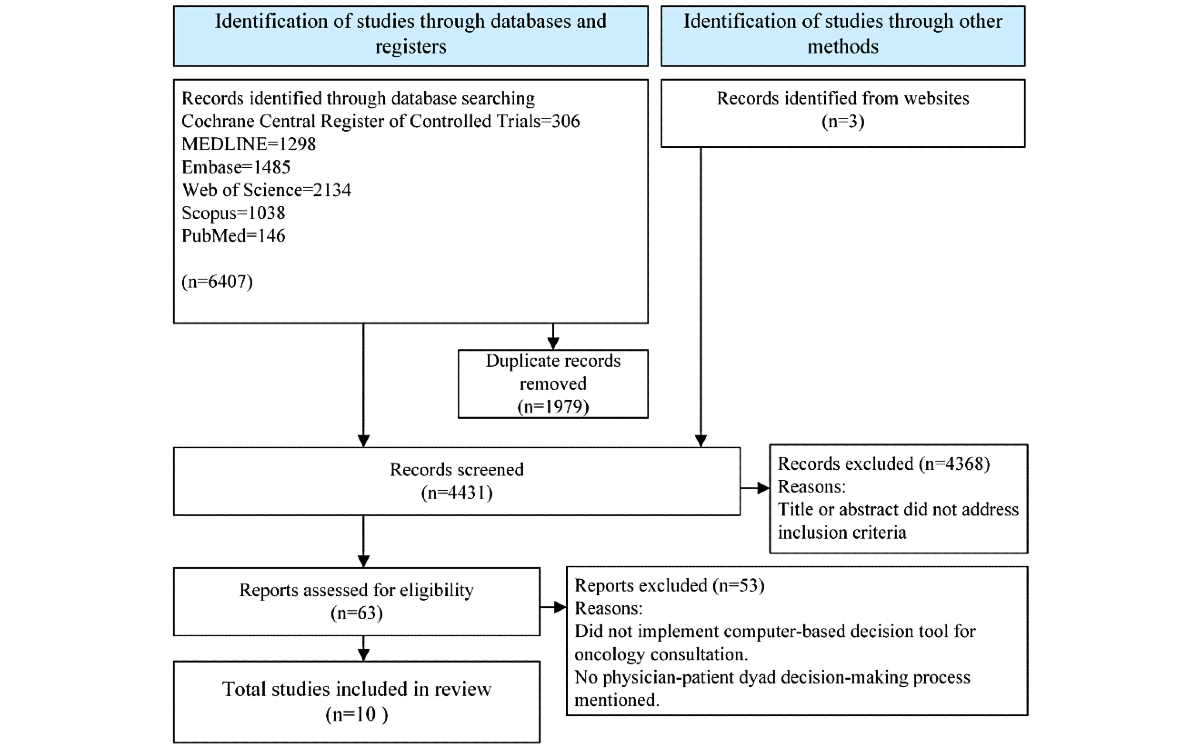
PRISMA (Preferred Reporting Items for Systematic Reviews and Meta-Analyses) 2020 flowchart of the study selection process and results.

The initial searches in the aforementioned databases retrieved 6407 articles (Figure 2). Browsing searches and inspections of reference lists and citations identified 3 additional articles. Of the 6407 articles retrieved through database search, 1979 (30.89%) duplicates were removed. Of the remaining total 4431 articles, 4368 (98.58%) were excluded after titles and abstracts were screened. Next, the full-text articles were assessed for eligibility, and of the 63 articles, 53 (84%) were excluded. A total of 10 studies were thus included in this review.

When the 10 selected studies for review were assessed by using the Cochrane risk-of-bias tools, they all exhibited some level of risk of bias. Of the 10 studies, 3 (30%) were randomized controlled trials [38,44,45], and 1 (10%) was a mixed-methods randomized study [41] (Multimedia Appendix 2, Table S1 [38,41,44,45]). All (4/4, 100%) the randomized studies included a high risk of bias because of the practices observed when assigning participants, adhering to the intervention, and accounting for missing outcome data. Of the 10 studies, 6 (60%) were nonrandomized studies (Multimedia Appendix 2, Table S2 [36,37,39,40,42,43]). Of these 6 nonrandomized studies, 1 (17%) [39] included a moderate risk of bias, whereas the remaining 5 (83%) [36,37,40,42,43] included serious risk of bias due to confounding [36,37,40], bias in selecting participants [43], bias in accounting for missing data, and measurement of outcomes [42].

Table 1 includes significant details gathered from the reviewed studies. Of the 10 studies, 6 (60%) were conducted in the United States, and 1 (10%) each was conducted in Canada, the Netherlands, Norway, and the United Kingdom. In all, 8 different computer-based decision tools were used across the 10 studies.

A summary of the identified computer-based decision tools from the review is provided in Table 2. The details include the name of the computer-based decision tool, country where each evaluation was conducted, categories of disease that were handled, types of decision that were settled, number of studies that were conducted for each computer-based decision tool, and bibliographical references. Of the 8 computer-based decision tools, 4 (50%) were evaluated for breast cancer consultations; 1 (13%) each for colorectal, prostate cancer, and cancer pain; and 1 (13%) for breast or colorectal cancer.

**Table 2:** Summary of 8 identified computer-based decision tools from 10 reviewed studies.

Name of computer-based decision tool	Country	Disease category	Type of decision	Number of studies	Reference
Adjuvant!	United States	Breast cancer	Take adjuvant chemotherapy or not	2	[44,45]
In-visit decision aid	United States	Breast cancer	Choose surgical option	2	[37,40]
Morgan	Canada	Breast cancer	Educate patients about adjuvant systemic therapy	1	[43]
TakeTheWind	United States	Breast cancer	Choose surgical option	1	[36]
SEER*CSC^a^	United States	Breast or colorectal cancer	Estimate patient prognosis	1	[42]
Openclinical	United Kingdom	Colorectal cancer	Take adjuvant chemotherapy or not	1	[41]
COMBAT	Norway	Cancer pain	Choose opioid dose and pain management option	1	[39]
Prostaat	Netherlands	Prostate cancer	Choose surgical and radiotherapy or no treatment	1	[38]

^a^SEER*CSC: Surveillance, Epidemiology, and End Results Cancer Survival Calculator.

### Factors Influencing Adoption of a Computer-Based Decision Tool

#### Levels of Impact

**Figure 3:**
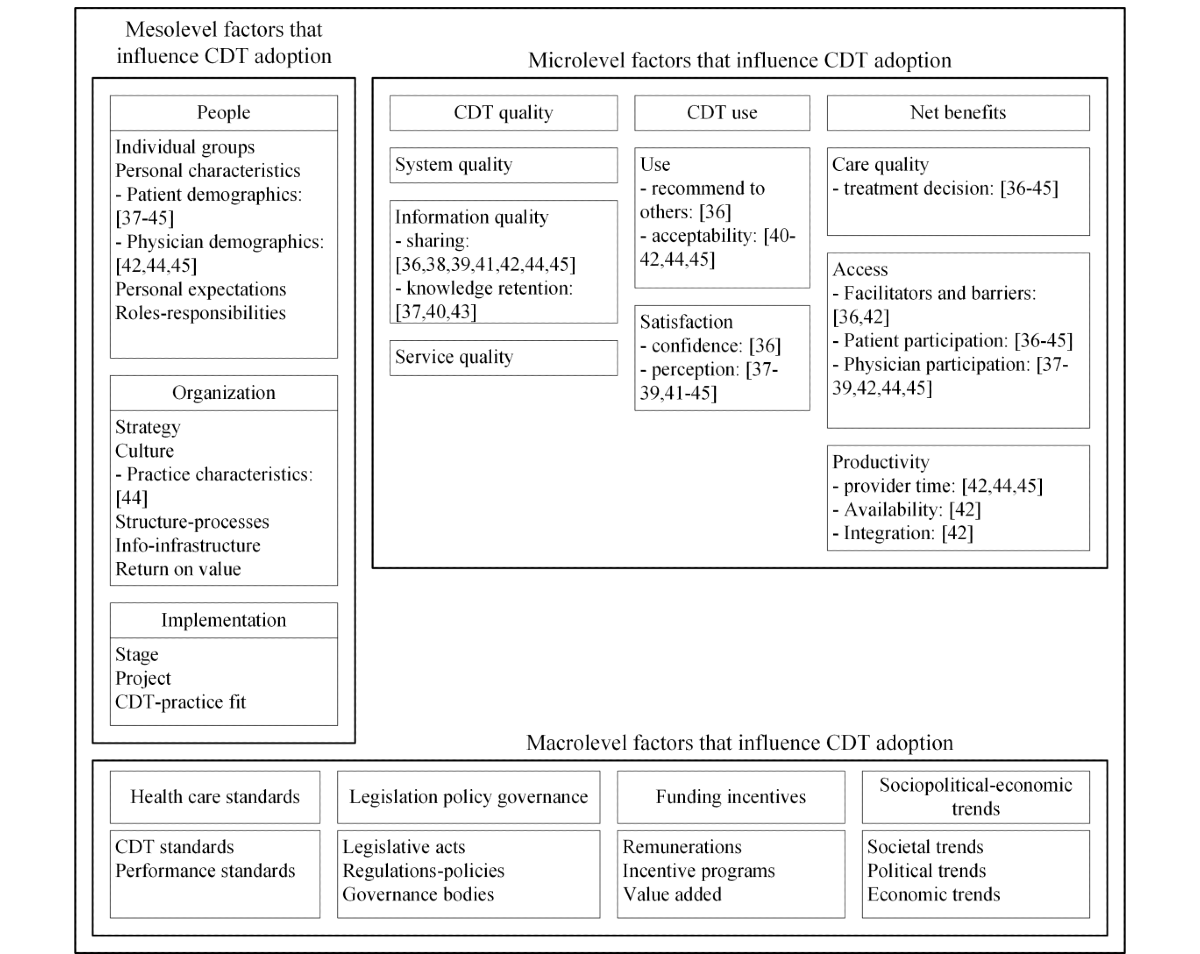
Micro-, meso-, and macrolevel factors that influence computer-based decision tool adoption [22]. CDT: computer-based decision tool.

The factors that influenced the adoption of computer-based decision tools during oncology consultations were identified from the 10 selected studies. An initial 16 distinct influential factors were collected from the review and mapped to the categories of the CAF as shown in Figure 3. Afterward, these 16 factors were expanded to show their levels of impact on adoption as shown in Multimedia Appendix 3 [36-45] and in the following sections [22].

#### Microlevel

##### Quality of System, Information, and Service

At the microlevel, no system or service quality factors were identified. However, information quality factors included information-sharing and knowledge retention. Transfer of information between patients and physicians was assessed by 30% (3/10) of the studies, which reported that patients retained a high level of treatment knowledge after consultations with physicians who used a computer-based decision tool [37,40,43]. Of the 10 studies, 5 (50%) assessed the level of information-sharing. Of these 5 studies, 1 (20%) found that 81.4% of the physicians considered the information provided by the computer-based decision tool useful [44], 2 (40%) reported that patients found the information about treatment options useful [36,41], and the remaining 2 (40%) reported that physicians did not use the information provided by the computer-based decision tool [39,42]. Of the remaining 5 studies, 1 (20%) reported that 65% of the patients read all information provided about treatment comparisons, and 71% of the patients indicated that they discussed the summary that was provided by the computer-based decision tool in consultation with their physicians [38]. A few physicians believed that some patients were made more anxious by the information, did not understand key information [44], were confused by the information provided, or felt that the information provided was conflicting [41]. In addition, some physicians did not value or benefit from the information provided by the computer-based decision tool [39].

##### Use and User Satisfaction

All 10 reviewed studies discussed use and user satisfaction. The use factors included recommendation and acceptability of use. Of the 10 studies, 1 (10%) [36] reported that when patients were introduced to the technology, 92% indicated that they liked it and would recommend its use to other patients. The feature that they liked the most was the *presence of helpful information*, followed by *ease of navigation* and *confidence in the treatment plan*. After consultations with physicians who used the technology, patients experienced a positive increase in confidence by an average of 0.8 points on a 10-point scale compared with when the technology was not used, and this was statistically significant [36]. However, the study also pointed out that some patients found navigating the technology difficult, disliked the use of tablet computers, and preferred written or printed material [36]. Similarly, another study (1/10, 10%) reported that 22% of the patients preferred consultations with paper-based decision tools [38]. In other cases, physicians provided patients with external access through web technologies to educate and prepare them for discussions about therapeutic choices during consultations. In these cases, other care providers such as nurses were also able to help by walking patients through the information provided by the technology and helped them increase their understanding of the benefits and risks of the different therapies on offer. Of the 10 studies, 4 (40%) reported that this practice was positively acceptable to both physicians and patients, although patients reportedly found the language of computer-based decision tools too complex [41,42,44,45]. Physicians found that their patients communicated better and engaged more in discussions. They felt that they were able to refine their understanding of their patients’ preferences, whereas patients felt that their perspectives were made clearer and reflected more accurately [44]. Patients’ satisfaction with consultations and clinic visits when computer-based decision tools were used was estimated to have a mean satisfaction score of 4.53 (SD 0.1) out of a maximum score of 5 [43]. However, of the 10 studies, 3 (30%) disclosed that computer-based decision tools did not improve therapeutic decision-making or found no statistically significant difference between decisions made using the technology and usual care and did not change physicians’ usual behavior [37,39,40].

##### Net Benefits in Terms of Care Quality, Access, and Productivity

Of the 10 studies, 8 (80%) referred to care quality factors as net benefits of computer-based decision tools. The studies [36-41,44,45] measured the proportion of patients who received various types of treatment. Siminoff et al [44] indicated that the difference in the proportion of patients receiving various types of therapy was statistically insignificant but stated that the adoption of computer-based decision tools during oncology consultations influenced 86.2% of the patients’ treatment decisions. The authors also declared that 84.6% of the patients in technology-assisted consultations accepted treatment compared with 89.5% of the patients in usual care. Furthermore, Peele et al [45] reported that only 58% of the women in consultations with technology accepted adjuvant therapy, an additional treatment to enhance the effectiveness of an initial medical treatment, compared with 87% of the women in usual care, and Yao et al [37] reported that 15.9% of the patients with low tumor severity in technology-assisted consultations accepted treatment compared with 24.6% in usual care. Similarly, Miles et al [41] reported that when technology was used in consultations, 11 out of 12 patients declined chemotherapy.

In contrast, of the 10 studies, 3 (30%) reported that patients in consultations with computer-based decision tools received more treatments than those in usual care. In a computer-based decision tool study for prostate cancer, 71% of the patients received treatment [38]. In a study for breast cancer treatment, 21.7% of the patients underwent surgery compared with 15.8% in usual care [37]. In addition, significantly more patients with high tumor severity chose adjuvant therapy in the computer-based decision tool group [45].

Of the 10 studies, 1 (10%) examined the effects of technology-assisted consultations on cancer pain intensity [39]. The authors observed no significant difference in pain intensity when technology was used compared with before its introduction. In addition, after 3 weeks of follow-up care, the authors noted that there was a lack of efficacy when the technology was used.

Of the 10 studies, 2 (20%) discussed access factors. The first study collected information on the facilitators and barriers to local adoption and implementation of a computer-based decision tool [42]. The study mentioned that the facilitators or barriers included existing channels, processes, and provider preferences. Users revealed that they did not access the technology because of lack of incentives or infrastructure, time, information about treatment, integration with the electronic health record system, availability of the technology on their desktops, and their own habits or preferences [42]. The second study produced a nonprioritized list of the facilitators and barriers to access [36]. The study identified that users needed to enter their username and password to log in, or they encountered technical issues every time they tried to use the technology; users had difficulty connecting wirelessly to the internet; and users were being provided information that they had already received on paper or during consultation [36].

Productivity factors covered the length of consultations. Of the 10 studies, 1 (10%) measured physicians’ productivity in terms of the effect of a computer-based decision tool on the length of consultations [44], and it found that an average of 5 minutes was added to the length of consultations.

#### Meso- and Macrolevels

Of the 10 studies, 9 (90%) identified patient demographics, 3 (30%) identified physician demographics, and 1 (10%) identified practice characteristics as mesolevel factors. However, there were no factors identified that explicitly influenced adoption at the mesolevel. At the macrolevel, there were no health care standards; legislations; policies; governance; funding incentives; or societal, political, or economic factors identified that explicitly influenced adoption.

### Summary of Key Findings

The results of this review showed that of the 8 identified computer-based decision tools, 4 (50%) were developed and studied in the United States, as shown in Table 2 [36,37,40,42,44,45]. Next, to determine whether a study was positive, negative, or neutral, the greater than or equal (≥) 50% rule, as cited in the study by Lau et al [22], was adopted. Consequently, of the 10 studies, 6 (60%) reported positive results for computer-based decision tools [37,38,41,43-45], whereas only 1 (10%) reported negative results [42]; 3 (30%) were neutral [36,39,40].

The CAF was extended to accommodate factors that influenced physicians’ propensity to adopt computer-based decision tools in oncology consultations. Of the 83 factors at the microlevel, 20 (24%) were identified as influential (Multimedia Appendix 3). Of these 20 factors, Textbox 1 reports 11 (55%) that were identified as positively affecting physicians, Textbox 2 reports 7 (35%) that negatively affected physicians, and Textbox 3 reports 2 (10%) that had no effect on physicians.

The studies did not explicitly provide evidence of meso- and macrolevel factors that influenced physicians’ propensity to adopt computer-based decision tools.

**The positive factors that influenced physicians’ propensity to adopt computer-based decision tools (N=11).**

**Factors that were identified as positively affecting physicians**

- Access
  Factor 1: treatment decisions were influenced by recommendations from physicians.
  Factor 2: information provided by the technology was given to patients by physicians.
  Factor 3: treatment information and the relationship with survival were included to facilitate conversation with patients.
  Factor 4: technology helped physicians to understand patients’ treatment preferences.
  Factor 5: information provided by the technology was useful to physicians.
  Factor 6: a copy of the information produced by the technology was used for reference during consultations.
- Information quality
  Factor 7: physician-patient communication about preferences and values was improved.
  Factor 8: physicians reviewed information provided by the technology with patients during consultations.
- Satisfaction
  Factor 9: physicians believed that patients became more engaged in discussion and understood the information.
- Use
  Factor 10: physicians reported that the technology was useful for their patients.
  Factor 11: the technology was used in routine practice in academic and community practices.

**The negative factors that influenced physicians’ propensity to adopt computer-based decision tools (N=7).**

**Factors that were identified as negatively affecting physicians**

- Access
  Factor 12: the technology did not provide all the information that the physicians wanted.
  Factor 13: the technology was not readily available on the physicians’ desktop.
  Factor 14: the technology was not integrated with the electronic health record.
- Information quality
  Factor 15: physicians did not take advantage of the information conveyed through the technology.
  Factor 16: physicians were not able to share information and treatment alternatives with their patients.
- Productivity
  Factor 17: the technology added 5 minutes to total consultation time.
- Satisfaction
  Factor 18: some physicians perceived that the technology made patients somewhat more anxious.

**The factors that showed that the use of computer-based decision tools had no effect on physicians’ propensity to adopt the technology (N=2).**

**Factors that were identified as not affecting physicians**

- Access
  Factor 19: no significant change in physicians’ behavior.
- Care quality
  Factor 20: no significant change in prescribed drug dosage between preintervention and intervention periods.

## Discussion

### Making Sense of the Adoption Success of Computer-Based Decision Tools in Oncology Consultations

This review has 3 aims: (1) to understand the different levels and periods of adoption of computer-based decision tools during oncology consultations across the world, (2) to identify the factors that influenced the adoption of the technology by physicians, and (3) to learn how to guide future implementation and adoption of the technology in the context of shared therapeutic decision-making during oncology consultations [48].

This review showed that the development and studies of computer-based decision tools were primarily conducted in North America and Europe in the last 16 years. Although 10 studies were specifically selected for review based on the topic of computer-based decision tools that were used by physicians in oncology consultations, only 60% (6/10) of the studies addressed some aspects of the perspectives of physicians. Most of the studies focused on patients’ views. Our findings of low adoption of computer-based decision tools converged with similar patterns in previous studies [49].

In all, 2 computer-based decision tools—Adjuvant! and an in-visit decision aid—were used across 40% (4/10) of the studies. Adjuvant! provided the strongest evidence of user satisfaction, information-sharing, care quality, and productivity measures. The in-visit decision aid was assessed for users’ perception, knowledge retention, and treatment decision. A summary of the 8 identified computer-based decision tools is provided in Table 2.

By extending the CAF to computer-based decision tools in oncology consultations, these findings suggest that of the 20 factors, there are 11 (55%) that can facilitate physicians to adopt the technology and 7 (35%) that can stifle adoption, whereas 2 (10%) may have no effect on physicians’ propensity to change and adopt the technology.

Along with helping physicians to understand their patients’ treatment preferences, computer-based decision tools enable physicians to refer to information and to provide treatment information and recommendations that are related to their patients’ survival. Some physicians used the technology in routine practice in academic and community practices to review information with patients during consultations. They believed that the technology is useful for their patients because their patients become more engaged in discussions and understood the information. Thus, the conversation between the physician and the patient was facilitated during consultations, and the patient-physician communication about preferences and values improved.

In contrast, some physicians perceived that computer-based decision tools made patients more anxious and added 5 minutes to their total consultation time. The study by Siminoff et al [44] gave the impression that an additional 5 minutes was insignificant. The effect, however, was subjective, depending on each physician’s expectation. For a 1-hour consultation, an additional 5 minutes may be acceptable. However, the impact of adding 5 minutes to a 10-minute consultation in usual care may become objectionable. Furthermore, when the technology does not provide all the information that physicians want, is not readily available on their desktop, or is not integrated with the electronic health record, then physicians are not able to take advantage of the information conveyed through the technology. Consequently, they are not able to share information and treatment alternatives with their patients.

The findings of this review advance our understanding of the extent to which computer-based decision tools have been successfully adopted in oncology consultations. The evidence suggests that there have been very few studies that address physicians’ propensity to adopt computer-based decision tools in routine oncology consultations. This review provides a starting point and direction for further investigations to incorporate computer-based decision tools in usual oncology consultations. This review also provides a guide and key lessons—as shown in Textboxes 1, 2, and 3—for the design and development of new computer-based decision tools. In addition, the review highlighted some important areas that need to be improved in future computer-based decision tools, such as integrated access with electronic medical records (Textbox 2). Some studies have reported negative outcomes with computer-based decision tools [50,51], whereas others have shown benefits [52]. In our review, of the 10 selected studies, 6 (60%) were positive, with only 1 (10%) being negative, whereas 3 (30%) were neutral. Consequently, the impact of computer-based decision tools on oncology consultations is unclear. Taken together, our findings and the findings of similar past studies [19-21,53-56] point to the need for further research in several dimensions of the CAF to uncover the value of computer-based decision tools in oncology practice.

Looking at Figure 3, it is obvious that the studies included in this review have addressed only a small set of factors among the numerous factors that could influence the adoption of computer-based decision tools in oncology consultations. Therefore, future studies will need to address additional dimensions at the meso- and macrolevels to gain a better understanding of what factors lead to successful implementation and adoption of computer-based decision tools in oncology consultations.

### Review Limitations

This systematic literature review includes some limitations. First, only 10 studies were included in this review because of the dearth of studies that addressed the issues with computer-based decision tools from the perspectives of physicians. Second, the literature search was conducted by only 1 reviewer, which could have introduced bias and limited the findings. Third, the selected studies for review included a high risk of bias. Furthermore, most of the studies were conducted at nontraditional cancer centers or at health care organizations affiliated with academic institutions, which limit generalization. Fourth, our review covered a wide range of health information systems’ issues, which might not have been explored sufficiently and fully explained. Future researchers should refine the search strategy to identify additional potentially relevant studies that may have been missed and allocate more reviewers to search the literature databases to minimize potential biases.

### Conclusions

In this review, we investigated the extent to which computer-based decision tools have been adopted in oncology consultations and physicians’ propensity to adopt the technology. The results of the investigation suggest that the adoption of computer-based decision tools in oncology consultations remains low. Of our 10 reviewed studies, 6 (60%) showed positive outcomes, whereas 1 (10%) showed negative outcomes, and 3 (30%) were neutral. To date, improvements have been made in communication and information-sharing between patients and physicians. However, unavailability of the information that physicians need, lack of access to the technology on physicians’ desktops, and lack of integration with existing electronic health record systems are some of the findings that stifle successful adoption. Therefore, this review shows that, in addition to improving communications between physicians and patients, technology is needed to streamline the flow of information that physicians need to better inform patients. Notwithstanding the 5 minutes that would be added to the overall time of consultations, this review indicates that it is possible to create leaner oncology practices by adopting computer-based decision tools. The technology would eliminate the need to track paper-based information, making the decision-making process more streamlined and eliminating the risk of missing hard-copy paperwork. Hence, in the long run, physicians would have more time to dedicate to their patients. As a result, patients may engage more in discussions during consultations, may be better informed, and they may be more apt to provide consent for treatment.

The CAF provides the capacity to make sense of complex multidimensional factors that influence the adoption of computer-assisted decision-making in oncology consultations. Furthermore, it provides a starting point as well as a sense of direction for research in the design and development of new computer-based decision tools. Thus, this review provides a set of key factors that need to be addressed to enhance the possibility of successfully implementing and adopting computer-based decision tools in oncology consultations. However, although the review shows that it is possible at the microlevel for patients and physicians to improve their communication by using computer-based decision tools, the effects of meso- and macrolevel factors remain understudied. It is therefore important to conduct additional studies in real-world oncology consultations to understand the impact of higher-level factors on physicians’ propensity to adopt computer-based decision tools.

## Appendix

Multimedia Appendix 1 Article search results and screening.

Multimedia Appendix 2 Risk of bias in individual randomized controlled trials and nonrandomized studies of interventions.

Multimedia Appendix 3 Datasheet of microlevel factors extracted from 10 reviewed studies.

## Abbreviations

CAF: Clinical Adoption Framework
PRISMA: Preferred Reporting Items for Systematic Reviews and Meta-Analyses
