# Supplementary material for: Computer-Based Decision Tools for Shared Therapeutic Decision-making in Oncology: Systematic Review
Source: JMIR Cancer. 2021 Oct 26;7(4):e31616. doi: 10.2196/31616 (PMC8579220; doi:10.2196/31616)
Supplement: Multimedia Appendix 1 [file cancer_v7i4e31616_app1.docx]

# **Multimedia Appendix 1**

Databases search strategy

EBM Reviews - Cochrane Database of Systematic Reviews 2005 to January 28, 2021.

| 1 | (internet* or web* or online* or onADJline* or computer* or software* or technology* or electronic* or digital*).m_titl. |
| --- | --- |
| 2 | (decision* or teaching*).m_titl. |
| 3 | (cancer* or neoplasm*).mp. [mp=title, abstract, full text, keywords, caption text] |
| 4 | 1 and 2 and 3 |
| 5 | limit 4 to full systematic reviews |

EBM Reviews - Cochrane Central Register of Controlled Trials December 2020.

| 1 | (internet* or web* or online* or onADJline* or computer* or software* or technology* or digital* or calculator*).mp. [mp=title, original title, abstract, mesh headings, heading words, keyword] |
| --- | --- |
| 2 | (decision* or teaching*).mp. [mp=title, original title, abstract, mesh headings, heading words, keyword] |
| 3 | (cancer* or neoplasm*).mp. [mp=title, original title, abstract, mesh headings, heading words, keyword] |
| 4 | (patient* and (physician* or oncologist*)).mp. [mp=title, original title, abstract, mesh headings, heading words, keyword] |
| 5 | 1 and 2 and 3 and 4 |

Ovid MEDLINE(R) ALL 1946 to February 04, 2021.

| 1 | (internet* or web* or online* or onADJline* or computer* or software* or technology* or calculator* or electronic* or digital*).mp. or exp Computers/ [mp=title, abstract, original title, name of substance word, subject heading word, floating sub-heading word, keyword heading word, organism supplementary concept word, protocol supplementary concept word, rare disease supplementary concept word, unique identifier, synonyms] |
| --- | --- |
| 2 | [decision* or teaching*).mp. or (exp Clinical Decision-Making/ or Clinical Decision-Making.mp. or (exp Clinical Decision Rules/ or Clinical Decision Rules.mp.) or (exp Decision Making/ or Decision Making.mp) or (exp Decision Making, Computer-Assisted/ or Decision Making, Computer-Assisted.mp.) or (exp Decision Support Systems. Clinical/ or Decision Support Systems. Clinical.mp.] or (exp Decision Support Systems, Management/ or Decision Support Systems, Management.mp.) or (exp Decision Support Techniques/ or Decision Support Techniques.mp.)) [mp=title, abstract, original title, name of substance word, subject heading word, floating sub-heading word, keyword heading word, organism supplementary concept word, protocol supplementary concept word, rare disease supplementary concept word, unique identifier, synonyms] |
| 3 | cancer* mp or (exp Neoplasms/ or Neoplasms.mp.) [mp=title, abstract, original title, name of substance word, subject heading word, floating sub-heading word, keyword heading word, organism supplementary concept word, protocol supplementary concept word, rare disease supplementary concept word, unique identifier synonyms] |
| 4 | patient* mp or (exp Patients/ or Patients.mp.) [mp=title, abstract, original title, name of substance word, subject heading word, floating sub-heading word, keyword heading word, organism supplementary concept word, protocol supplementary concept word, rare disease supplementary concept word, unique identifier, synonyms] |
| 5 | oncologist*.mp or (exp Oncologists/ or Oncologists.mp.) or (exp Medical Oncology/ or Medical Oncology.mp. or (exp Oncology Service, Hospital/ or Oncology Service, Hospital.mp.) or (exp Radiation Oncology/ or Radiation Oncology.mp.) or (exp Surgical Oncology/ or Surgical Oncology.mp.)) or (exp Physicians/ or Physicians.mp.) or physician*.mp. [mp=title, abstract, original title, name of substance word, subject heading word, floating sub-heading word, keyword heading word, organism supplementary concept word, protocol supplementary concept word, rare disease supplementary concept word, unique identifier, synonyms] |
| 6 | 1 and 2 and 3 and 4 and 5 |
| 7 | limit 6 to (humans and "all adult (19 plus years)") |

Embase Classic+Embase 1947 to 2021 February 04.

| 1 | (internet* or web* or online" or onADJline* or computer* or software* or technology* or digital* or electronic* or calculator*).mp. [mp=title, abstract, heading word, drug trade name, original title, device manufacturer, drug manufacturer, device trade name, keyword, floating subheading word, candidate term word] |
| --- | --- |
| 2 | (decision* or teaching*).mp. [mp=title, abstract, heading word, drug trade name, original title, device manufacturer, drug manufacturer, device trade name, keyword, floating subheading word, candidate term word] |
| 3 | [cancer* or neoplasm*).mp. [mp=title, abstract, heading word, drug trade name, original title, device manufacturer, drug manufacturer, device trade name, keyword, floating subheading word, candidate term word] |
| 4 | (patient* and (physician* or oncologist*)).mp. [mp=title, abstract, heading word, drug trade name, original title, device manufacturer, drug manufacturer, device trade name, keyword, floating subheading word, candidate term word] |
| 5 | 1 and 2 and 3 and 4 |
| 6 | limit 5 to (human and adult <18 to 64 years>) |

Web of Science 1900 to 2021.

| 1 | ALL FIELDS: (internet* or web* or online* or "on line" or computer* or software* or technology* or digital* or calculator* or electronic*) AND ALL FIELDS:  (decision* or teaching*) AND ALL FIELDS: (cancer* or neoplasm*) AND ALL FIELDS: (patient*) AND ALL FIELDS: (physician* or oncologist*)  lndexes=SCI-EXPANDED, SSCI, A&HCl, CPCI-S, CPCI-SSH, ESCI, CCR-EXPANDED, 1C Timespan=All years |
| --- | --- |

Scopus 1969 to 2021.

| 1 | (TITLE-ABS-KEY (internet* OR web* OR online* OR “on line” OR computer* OR software* OR technology* OR electronic* OR digital* OR calculator* AND TITLE-ABS-KEY (decision* OR teaching* ) AND TITLE-ABS-KEY (cancer* OR neoplasm*) AND TITLE-ABS-KEY (patient*) AND TITLE-ABS-KEY (physician* OR oncologist*)) AND (LIMIT-TO ( EXACTKEYWORD, "Adult")) |
| --- | --- |

PubMed 1991 to 2021.

| 1 | Search: (("decision aid"[Title])) AND (therapy[Title] OR mastectomy [TITLE] OR chemotherapy [TITLE] OR treatment[Title]) |
| --- | --- |
