# Supplementary material for: Computer-Based Decision Tools for Shared Therapeutic Decision-making in Oncology: Systematic Review
Source: JMIR Cancer. 2021 Oct 26;7(4):e31616. doi: 10.2196/31616 (PMC8579220; doi:10.2196/31616)
Supplement: Multimedia Appendix 2 [file cancer_v7i4e31616_app2.docx]

# **Multimedia Appendix 2**

Table S1: Risk of Bias in individual randomized controlled trial. Low = low risk of bias; High = high risk of bias; Unclear = unclear or not reported.

| **Author (s), year** | **Randomization** | **Assignment to intervention** | **Adhering to intervention** | **Missing outcome data** | **Measurement of the outcome** | **Selection of the reported result** | **Overall risk of bias** | **Reference** |
| --- | --- | --- | --- | --- | --- | --- | --- | --- |
| (Cuypers M, et al., 2019) | Low | High | High | High | High | Unclear | High | [38] |
| (Miles, Chronakis, Fox, & Mayer, 2017) | High | High | High | High | High | Unclear | High | [41] |
| (Siminoff, Gordon, Silverman, Budd, & Ravdin, 2006) | High | High | High | High | Unclear | High | High | [44] |
| (Peele, Siminoff, Xu, & Ravdin, 2005) | High | High | High | High | Low | Unclear | High | [45] |

Table S2: Risk of Bias in non-randomized studies of interventions. Low = low risk of bias; Moderate = moderate risk of bias; Serious = serious risk of bias; Critical = critical risk of bias; NI = no information available.

| **Author (s), year** | **Due to confounding** | **Selection of participants** | **Classification of intervention** | **Deviations from intervention** | **Missing data** | **Measurement of outcomes** | **Selection of reported result** | **Overall risk of bias** | **Reference** |
| --- | --- | --- | --- | --- | --- | --- | --- | --- | --- |
| (Wyatt, Jenkins, Plevak, Venegas Pont, & Pruthi, 2019) | Serious | Moderate | Moderate | Moderate | Moderate | Moderate | Moderate | Serious | [36] |
| (Yao, et al., 2019) | Serious | Moderate | Moderate | Moderate | Moderate | Moderate | Moderate | Serious | [37] |
| (Raj, Brunelli, Klepstad, & Kaasa, 2017) | Moderate | Moderate | Moderate | Moderate | Moderate | Moderate | Moderate | Moderate | [39] |
| (Yao, et al., 2017) | Serious | Moderate | Moderate | Moderate | Moderate | Moderate | Moderate | Serious | [40] |
| (Henton, Gaglio, Cynkin, Feuer, & Rabin, 2017) | NI | Moderate | Moderate | Moderate | Serious | Serious | NI | Serious | [42] |
| (Morgan, Laing, McCarth, McCrate, & Seal, 2015) | NI | Serious | Moderate | Moderate | Moderate | Moderate | Moderate | Serious | [43] |
