# Supplementary material for: Computer-Based Decision Tools for Shared Therapeutic Decision-making in Oncology: Systematic Review
Source: JMIR Cancer. 2021 Oct 26;7(4):e31616. doi: 10.2196/31616 (PMC8579220; doi:10.2196/31616)
Supplement: Multimedia Appendix 3 [file cancer_v7i4e31616_app3.docx]

# **Multimedia Appendix 3**

Table S3: Datasheet of microlevel factors extracted from ten reviewed studies.

| **Clinical Adoption Framework**  ***Microlevel dimension*** | **Measure** | **Impact by measure** | **Factors that influenced computer-based decision tool adoption** | **Country** | **Year** | **Reference** |
| --- | --- | --- | --- | --- | --- | --- |
| Information quality | Sharing | Positive | Patients preferred shared decision making. | US | 2019 | [36] |
| Information quality | Sharing | Negative | Patients preferred written or printed material. | US | 2019 | [36] |
| Information quality | Sharing | Negative | Patients disliked tablet computers. | US | 2019 | [36] |
| Use | Recommend use to others | Positive | Patients were encouraged to recommend use of the technology to other patients when it was used as part of their consultations. | US | 2019 | [36] |
| Satisfaction | Confidence | Positive | Patients’ levels of confidence in decision-making were increased when the technology was used as part of their consultations. | US | 2019 | [36] |
| Care quality | Treatment decision | Positive | Patients preferred to be engaged in shared decision making when making treatment decisions. | US | 2019 | [36] |
| Access | Barriers | Negative | An active internet connection was needed. | US | 2019 | [36] |
| Access | Barriers | Negative | Users needed to log in with a username and password each time the application was accessed. | US | 2019 | [36] |
| Access | Barriers | Negative | Users had trouble navigating the technology. | US | 2019 | [36] |
| Access | Barriers | Negative | Users had trouble accessing the technology. | US | 2019 | [36] |
| Access | Patient participation | Positive | Patients’ level of confidence increased when the technology was used in conjunction with clinical consultations. | US | 2019 | [36] |
| Access | Patient participation | Positive | When the technology was used as part of interdisciplinary consultations, patients expressed higher levels of confidence post-intervention. | US | 2019 | [36] |
| Information quality | knowledge retention | Positive | The technology increased patients’ knowledge thus decreased decisional conflicts. | US | 2019 | [37] |
| Satisfaction | Perception | Positive | Patients’ decisional satisfaction improved. | US | 2019 | [37] |
| Care quality | Treatment decision | Positive | Patients had less surgery. | US | 2019 | [37] |
| Care quality | Treatment decision | Neutral | Patients’ anxiety, distress, fear, quality of life and concerns regarding body image were unchanged compared with usual care. | US | 2019 | [37] |
| Access | Patient participation | Positive | Patients had more discussions regarding their treatment with surgeons. | US | 2019 | [37] |
| Access | Physician participation | Positive | Physicians’ recommendations can influence treatment decisions. | US | 2019 | [37] |
| Information quality | Sharing | Positive | Physicians-patient communication about preferences and values was improved. | Netherlands | 2019 | [38] |
| Information quality | Sharing | Positive | 65% of patients read all information about treatment comparisons. | Netherlands | 2019 | [38] |
| Information quality | Sharing | Positive | 78% of patients indicated that the format of the technology was preferred. | Netherlands | 2019 | [38] |
| Satisfaction | Perception | Negative | 22% of patients preferred a paper format. | Netherlands | 2019 | [38] |
| Care quality | Treatment decision | Positive | 71% of users indicated a treatment preference. | Netherlands | 2019 | [38] |
| Access | Patient participation | Positive | 71% of patients indicated that the summary provided by the technology was discussed with their physician during consultation. | Netherlands | 2019 | [38] |
| Access | Physician participation | Positive | Physicians provided access to the information generated by the technology to patients. | Netherlands | 2019 | [38] |
| Information quality | Sharing | Negative | Physicians did not take advantage of the information conveyed through the technology. | Norway | 2017 | [39] |
| Care quality | Treatment decision | Neutral | Only 10.4% of the patients started with new pain medication. Not significantly different compared to the pre-intervention period. | Norway | 2017 | [39] |
| Care quality | Treatment decision | Neutral | Only 12% of patients changed existing medication. Not significantly different compared to the pre-intervention period. | Norway | 2017 | [39] |
| Care quality | Treatment decision | Neutral | No improvement in pain management. | Norway | 2017 | [39] |
| Care quality | Treatment decision | Neutral | No differences in pain intensity scores after introducing the technology. | Norway | 2017 | [39] |
| Care quality | Treatment decision | Negative | A lack of efficacy with the technology was reported. | Norway | 2017 | [39] |
| Care quality | Treatment decision | Negative | No differences in treatment after introducing the technology. | Norway | 2017 | [39] |
| Care quality | Treatment decision | Neutral | In the pre-intervention period, physicians changed the dose of opioids in 18.8% of the patients compared to 21.6% of the patients in the intervention period. | Norway | 2017 | [39] |
| Access | Physician participation | Neutral | No significant change in physicians’ behaviour. | Norway | 2017 | [39] |
| Information quality | Knowledge retention | Positive | Higher knowledge levels in the technology patient group than in usual care group. | US | 2017 | [40] |
| Information quality | Knowledge retention | Positive | The proportion of patients who answered questions correctly was higher in the technology cohort. This was statistically significant. | US | 2017 | [40] |
| Use | Acceptability | Neutral | No impact on choice of surgical treatment. Same proportion of patients underwent their preferred treatment between the technology and usual care groups. | US | 2017 | [40] |
| Care quality | Treatment decision | Neutral | There was no significant difference in treatment preference between the technology and usual care groups. | US | 2017 | [40] |
| Access | Patient participation | Neutral | Patients’ worry and anxiety were assessed. No significant difference between the technology and usual care groups. | US | 2017 | [40] |
| Information quality | Sharing | Negative | Needed improved presentation of information. | UK | 2017 | [41] |
| Information quality | Sharing | Negative | Lack of understanding of key information among some patients. | UK | 2017 | [41] |
| Information quality | Sharing | Positive | Information about the risk of recurrence was helpful. | UK | 2017 | [41] |
| Information quality | Sharing | Positive | Most patients were happy that they understood the meaning of the risk information provided by the technology. | UK | 2017 | [41] |
| Information quality | Sharing | Positive | Graphical representation of information increased clarity. | UK | 2017 | [41] |
| Information quality | Sharing | Negative | Some conflicting feelings about the information were reported. | UK | 2017 | [41] |
| Use | Acceptability | Positive | Patient acceptance was high. | UK | 2017 | [41] |
| Satisfaction | Perception | Positive | Patients perceived that the technology helped them communicate with their physicians and make decision. | UK | 2017 | [41] |
| Care quality | Treatment decision | Positive | Patients understood that the treatment goal was to decrease the risk of disease recurrence. | UK | 2017 | [41] |
| Care quality | Treatment decision | Negative | The figures about disease recurrence or progression were confusing. | UK | 2017 | [41] |
| Access | Patient participation | Positive | It was helpful for patients to see an illustration of the net benefit of therapy. | UK | 2017 | [41] |
| Information quality | Sharing | Negative | Physicians were not able to share information and treatment alternatives with their patients. | US | 2017 | [42] |
| Use | Acceptability | Positive | Patients felt it was easy to navigate and enter data. | US | 2017 | [42] |
| Use | Acceptability | Negative | The language and terminology used limited usage of the technology. | US | 2017 | [42] |
| Satisfaction | Perception | Positive | Patients liked the graphical representation of results that best meet their needs and ability to understand. | US | 2017 | [42] |
| Care quality | Treatment decision | Negative | There was a lack of treatment options. | US | 2017 | [42] |
| Access | Barriers | Negative | Physicians reported that the technology did not provide all the information that they wanted. | US | 2017 | [42] |
| Access | Barriers | Negative | Physicians reported that the technology was not readily available on their desktops. | US | 2017 | [42] |
| Access | Barriers | Negative | Physicians reported that the technology was not integrated with electronic health records. | US | 2017 | [42] |
| Access | Facilitator | Positive | Physicians felt it was important to include treatment information and the relationship with survival to facilitate conversation with their patients. | US | 2017 | [42] |
| Access | Patient participation | Positive | The technology helped patients to prepare for conversations with their health care team. | US | 2017 | [42] |
| Access | Physician participation | Negative | There was confusion about how to navigate the technology. | US | 2017 | [42] |
| Productivity | Time consuming | Negative | Data entry was found to be time consuming. | US | 2017 | [42] |
| Information quality | Knowledge retention | Positive | Knowledge retention after clinic visit was found to be high. | Canada | 2015 | [43] |
| Satisfaction | Perception | Positive | Patient satisfaction with consultation and clinic visit was high. | Canada | 2015 | [43] |
| Satisfaction | Perception | Positive | Patients were satisfied with tablet-based information. | Canada | 2015 | [43] |
| Care quality | Treatment decision | Positive | Patients identified that the intent of therapy was to reduce risk of disease recurrence, not to eliminate relapse. | Canada | 2015 | [43] |
| Access | Patient participation | Positive | Patients were comfortable with the technology. | Canada | 2015 | [43] |
| Information quality | Sharing | Positive | Patients found that the information provided by the technology was more influential on decisions. | US | 2006 | [44] |
| Use | Acceptability | Positive | The technology was found more useful than a paper pamphlet. | US | 2006 | [44] |
| Use | Acceptability | Positive | Physicians reported that the technology was useful for their patients. | US | 2006 | [44] |
| Satisfaction | Perception | Positive | Physicians believed that patients became more engaged in discussion and understood the information. | US | 2006 | [44] |
| Satisfaction | Perception | Negative | Some physicians perceived that the technology made patients somewhat more anxious. | US | 2006 | [44] |
| Care quality | Treatment decision | Positive | The technology influenced most patients when making their treatment decisions. | US | 2006 | [44] |
| Care quality | Treatment decision | Positive | Patients in the technology group took treatment less frequently that those in the control group. | US | 2006 | [44] |
| Access | Patient participation | Positive | Most patients reported that the technology was easy to understand. | US | 2006 | [44] |
| Access | Physician participation | Positive | Physicians reported that the technology helped them to understand patients’ treatment preferences. | US | 2006 | [44] |
| Access | Physician participation | Positive | Physicians reported that the information provided by the technology was useful to them. | US | 2006 | [44] |
| Productivity | Time consuming | Negative | Physicians reported that the technology added five minutes to total consultation time. | US | 2006 | [44] |
| Information quality | Sharing | Positive | Physicians reviewed the information provided by the technology with patients during consultations. | US | 2005 | [45] |
| Use | Acceptability | Positive | Physicians used the technology in academic and community practices. | US | 2005 | [45] |
| Care quality | Treatment decision | Positive | Patients with low tumor severity in the technology group rejected treatment significantly more often. | US | 2005 | [45] |
| Care quality | Treatment decision | Positive | Patients with high tumor severity in the technology group were more likely to choose treatment. | US | 2005 | [45] |
| Access | Patient participation | Positive | A standardized presentation of information was used with patients. | US | 2005 | [45] |
| Access | Physician participation | Positive | Physicians used a copy of the information produced by the technology for reference during consultations. | US | 2005 | [45] |
